# Current Perspectives on Cognitive Diversity

**DOI:** 10.3389/fpsyg.2016.00509

**Published:** 2016-04-12

**Authors:** Andrea Bender, Sieghard Beller

**Affiliations:** Department of Psychosocial Science, Faculty of Psychology, University of BergenBergen, Norway

**Keywords:** culture, universals and diversity, perception and categorization, numerical cognition, counting, explanatory frameworks

## Abstract

To what extent is cognition influenced by a person’s cultural background? This question has remained controversial in large fields of the cognitive sciences, including cognitive psychology, and is also underexplored in anthropology. In this perspective article, findings from a recent wave of cross-cultural studies will be outlined with respect to three aspects of cognition: perception and categorization, number representation and counting, and explanatory frameworks and beliefs. Identifying similarities and differences between these domains allows for general conclusions regarding cognitive diversity and helps to highlight the importance of culturally shaped content for a comprehensive understanding of cognition.

## Introduction

The question of the extent to which, if at all, cognition may be influenced by a person’s cultural background has remained controversial in large fields of the cognitive sciences, including cognitive psychology, and chapters on this issue remain absent from cognitive psychology textbooks. In fact, the majority of cognitive scientists do not even consider it a relevant question to begin with. This is largely due to some of the core assumptions on which cognitive science was originally built (e.g., Flanagan, 1991), according to which cognition itself is taken as universal, conceptualized as information processing based on knowledge representations. Only its *content*, such as people’s beliefs, has been widely conceived of as susceptible to cultural influences, and though initially regarded as a core issue for cognitive science (Norman, 1980), beliefs have been disregarded in the field at large (Bender et al., 2010; Bender and Beller, 2011b).

Interestingly, a similar reluctance to engage in cross-cultural research can be observed among many of those inherently interested in culture, that is, cultural anthropologists, but for entirely different reasons (Bloch, 2012). Besides epistemological disagreements and a somewhat underdeveloped interest in cognition, one of their main reasons for not looking into the possible cultural influences on cognition may have been the concern with what the outcome of cultural comparisons would imply. This concern, which weighs particularly heavily in light of the colonial, imperialistic, and racist periods not only in Euro-American history but also in Euro-American science, has prevented most anthropologists from regarding cross-cultural comparisons as a passable route. While this attitude is understandable, its consequences are detrimental, as the other cognitive sciences were left believing that their universalistic assumptions are valid. Disregarding the potential and actual diversity in cognition not only fails to do justice to those who are of concern to anthropologists, but also renders an inaccurate account of how the vast majority of humankind perceives, memorizes, or reasons.

Voices have been raised in criticism of this neglect (e.g., Arnett, 2008; Beller et al., 2012), and specifically the article on the “weirdest people” (Henrich et al., 2010) generated an upsurge of interest in cross-cultural studies^1^. Here, we intend to take stock of what has been achieved since then in three exemplary domains, in order to derive general conclusions on what we will be forfeiting if we do not take the cultural diversity of cognition more seriously.

## Cognitive Diversity in Three Domains

Partly in line with the emphasis in current research, this overview focuses on three exemplary aspects of cognition: perception and categorization, number representation and counting, and explanatory frameworks and beliefs.

### Perception and Categorization

Perception is the most basic process of gaining information, be it visual information about shape or color, acoustic information about words and other sounds, or olfactory information about smells. Our sensory apparatus receives signals, preprocesses them, and forwards them until, given sufficient attention, they become subject to further processing. Even if perception itself is often conceived of as distinct from cognition proper, its output is co-constructed in a joint bottom–up/top–down manner with the aid of higher-order cognitive processing, including those processes related to action (Witt, 2011), and hence may be affected by cultural factors (e.g., Nisbett and Miyamoto, 2005).

A particularly impressive example of both the non-objective nature of perception and its susceptibility to culture is the *Müller-Lyer illusion.* This illusion, in which two equally long lines appear to be of different lengths depending on the visual context, is robust and persists even after explicit instruction. And yet, of the 16 groups investigated in a cross-cultural study, US-American participants most strongly overestimated the difference between the lines, while this discrepancy was almost absent among hunter-gatherers in southern Africa (Segall et al., 1963, 1966; for other examples see also Ahluwalia, 1978; Sekiyama, 1997). Notably, this case of cultural differences in an ostensibly hard-wired illusion—although already reported in a high impact journal in the 1960s—only reached prominence after being showcased by Henrich et al. (2010). According to the most frequently cited explanation, the occurrence and extent of this illusion depends on the prevalence of rectangular shapes in people’s environment, and hence on the input to which the perceptual system is continuously trained and calibrated.

A more explicit effect of culture is discussed for *color discrimination*. Here, the question focuses on whether perception is affected not by environmental stimuli themselves, but by the color lexicon—that is, by a cognitively accessible, classificatory system; in this sense, perception is closely related to categorization (for excellent reviews on culture and categorization especially in the biological domain, see Berlin, 1992; Medin and Atran, 1999, 2004; Atran and Medin, 2008; Ojalehto and Medin, 2015). Although findings are more controversial for color categorization than for biological categorization (Regier et al., 2005; Roberson et al., 2005), they seem to confirm that, once language comes into play, color perception is indeed tinged (Regier and Kay, 2009). Similar findings are reported also for other domains (Malt and Majid, 2013; Majid and Van Staden, 2015) and even for sense modalities like olfaction, which have long been neglected by Western science as underdeveloped and coarse-grained (partly due to the coarse-grained taxonomy on smells in Western languages such as English). Recent studies on *olfaction* among hunter-gatherer groups in Southeast Asia, in contrast, furnished evidence for the capability of human languages to encode smell qualities in a much more fine-grained, coherently structured manner (Majid and Burenhult, 2014; Wnuk and Majid, 2014).

Language and cultural metaphors may also affect the perception and reproduction of other abstract experiences such as those pertaining to *time* (overviews in Núñez and Cooperrider, 2013; Bender and Beller, 2014b; Sinha and Gärdenfors, 2014) or *musical pitch*. A series of experiments on the latter revealed that speakers of languages in which pitch is habitually described in terms of height (such as English or Dutch) are affected by height-related stimuli when reproducing pitch, whereas speakers of languages in which pitch is described in terms of thickness (such as Farsi) are affected by thickness-related stimuli—even in entirely non-linguistic, psychophysical tasks (Dolscheid et al., 2013). While shaped by linguistic habits, these space-pitch associations also build on pre-existing associations already found in pre-linguistic infants (Dolscheid et al., 2014).

A cognitive process closely linked to perception is *attention*, by which awareness is directed to some segments in the stream of incoming signals. It has been known for some time that attention itself may also be susceptible to cultural influences, with North Americans tending to be less attentive to contextual information than East Asians (Nisbett and Masuda, 2003; Masuda and Nisbett, 2006), and especially so when asked to construct narratives of their observation (Senzaki et al., 2014). Attention can also, in line with cultural predispositions, be more or less explicitly directed to the quasi-perceptual experiences in hallucinations (Luhrmann et al., 2015) or dreams (Glaskin, 2011, 2015), guiding their interpretation and even the way in which people deal with them.

Attention is not only essential for what we become aware of, but partly also for how we integrate this new information—or how we express it through, for instance, artistic visualization. This was indicated in a study with three cultural groups in northern Siberia (Istomin et al., 2014); in each of the groups, both perceptual tests and artistic styles were based on the same perceptual tendencies, but these tendencies differed between groups, in line with differences in patterns of social and environmental interaction.

### Number Representation and Counting

A broad range of cognitive processes contributes to what can be called numerical cognition (Gaber and Schlimm, 2015), which is assumed to be based on three systems: two core systems for parallel individuation and approximate magnitude, which are available in human infants and non-human species, and a uniquely human, symbolic system implemented as counting sequence (Feigenson et al., 2004). The paramount importance of the symbolic system for human numerical cognition has been demonstrated among groups in Amazonia that use no or only imprecise number words (Gordon, 2004; Pica et al., 2004), Nicaraguan home-signers (Spaepen et al., 2011), and even US-American students when prevented from accessing the counting sequence (Frank et al., 2012).

If we disregard the availability of a symbolic system, it would appear at first glance that numerical cognition does not seem to allow for much variance. After all, numbers map onto objective and discrete quantities that are identical the world over, and the same seems to hold for basic arithmetic, if not advanced algebraic operations. And yet, even the manner in which the non-symbolic intuition on approximate magnitude is cognitively represented—namely with the characteristics of a *mental number line* (Dehaene, 2003; Dehaene et al., 2008)—is subject to cultural variability (Bender and Beller, 2011a). Factors contributing to this variability include, for instance, writing direction and finger counting habits, resulting in the reversal of the originally left-to-right orientation (Dehaene et al., 1993; Fischer and Brugger, 2011; Shaki and Fischer, 2014). A number line appears to be lacking entirely in participants who have not received any formal school education (Núñez, 2011; Núñez et al., 2012), suggesting that this line is more likely a product of the cultural habit of measuring with rulers than the result of a preexisting neural structure.

Cultural variability is amplified substantially when we turn to the *symbolic system*, particularly with regard to how the counting sequence itself is represented and structured, be it by number words, written notations, body parts, or otherwise external manners such as tallies or knotted strings. Each of these representations comes with (culture-)specific properties, which then affect how numerical information is cognitively represented and processed. This has been described and analyzed extensively for notational systems (Nickerson, 1988; Zhang and Norman, 1995; Chrisomalis, 2004; Schlimm and Neth, 2008; Widom and Schlimm, 2012), but also for body-based systems (Domahs et al., 2010; Beller and Bender, 2011; Bender and Beller, 2012) and even verbal systems (e.g., Miura et al., 1993; Bender et al., 2015b).

The key argument is that the properties of such a cognitive tool affect how it is used. The more regular a counting system is, for instance, the easier it is to learn and handle (Fuson and Kwon, 1991; Miura et al., 1993; Miller et al., 1995). The more compact representations it affords (e.g., due to larger counting units or smaller base size), the less cognitive load it will generate during mental arithmetic (Bender et al., 2015b). And when harnessing binary patterns, burdensome calculations can be replaced by simple transformations (Bender and Beller, 2014a, 2016b). All these system properties, along with their cognitive implications, vary substantially across languages and cultures, thus generating considerable potential for diversity in numerical cognition.

It should be stressed, however, that cognitive tools do not dictate cognitive behavior. Even though they may provide the foundation from which we reach further, we can steer the development and change these tools if they turn out to be insufficient (Beller and Bender, 2008), as attested to particularly by the history of numerical notations. Diachronic analyses of changes over time are thus an important addition to cross-cultural comparisons (Haidle, 2014; Iliev and Ojalehto, 2015).

### Explanatory Frameworks and Beliefs

The possibility that culture may affect cognition is perhaps most easily acknowledged for those aspects of cognition that are involved in people’s attempts to understand and explain properties, relations, or events. It is arguably here where culturally shaped beliefs most likely come to bear. How such beliefs, and patterns of reasoning from them, may affect causal attributions has been explored in a recent research topic (Beller et al., 2014) and summarized elsewhere (Bender and Beller, 2016a). Here, we focus on the question of whether cultural differences in expertise may affect explanatory depth, and discuss the implications that culture-specific explanatory frameworks may have for other domains of cognition, and the degree to which diverging belief systems can co-exist.

The relevance of *expertise* for explanations and reasoning has long been established, as has the fact that this expertise may be a product of culturally shaped behavioral and cognitive patterns (Medin and Atran, 2004; Atran and Medin, 2008). This is observable at an early age and to the extent that it shapes the course of cognitive development. But the impact of culture goes beyond simply pre-structuring experiences and hence expertise—it also conveys an *interpretative framework* for what one experiences (e.g., Bang et al., 2007; Bang and Medin, 2010; Ojalehto et al., 2013). Both of these effects were observed in a study comparing Menominee children from a Native American reservation and children from both a rural and an urban Euro-American population. Only the urban children displayed an anthropocentric perspective (assumed to be fundamental in developmental psychology) that prioritizes humans as the source for inferences, whereas the two rural groups were guided by their expertise on the natural environment. The two rural groups, on the other hand, differed in the degree to which they considered the relation between humans and non-human species as symmetric—a difference grounded in their respective cultural background (Ross et al., 2003).

More recently, the ramifications of such cultural epistemologies were studied in greater depth in a series of studies comparing US-Americans and Ngöbe from Panama (Ojalehto et al., 2015; and see Ojalehto and Medin, 2015). These two groups differ in their epistemological orientation to nature, which predisposes them to a distinct perspective on other species and their interrelations. Compared to the US-Americans, Ngöbe participants focus more on interconnectedness than individualization, and acknowledge social agency in non-human species to a greater extent. As a consequence, they are also more likely to consider non-human species as being capable of intentional communication and morality, and to interpret a given ecological relationship as based on cooperation rather than competition (Ojalehto et al., 2015).

Importantly, beliefs do not always, and not necessarily, generate coherent belief systems, and a growing number of studies are devoted to the conditions under which *conflicting beliefs* can co-exist. Starting off with the classical anthropological example of how Azande explain the collapse of a granary alternatively by termite infestation and witchcraft (Evans-Pritchard, 1937; and see Widlok, 2014), cognitive scientists have taken up this issue by exploring how people reconcile alternative beliefs for issues such as the origin of species, illness, death, or acts of wrong-doing (Astuti et al., 2004; Legare and Gelman, 2008; Hagmayer and Engelmann, 2014; Astuti and Bloch, 2015; Moya et al., 2015; Watson-Jones et al., 2015). The focus in these studies is directed toward “natural” vs. “supernatural” explanations and toward the modes through which such occasionally conflicting explanations are reconciled. According to a recently proposed model (Legare et al., 2012), the two types of explanations can be combined in one of three manners, resulting in target-dependent thinking, synthetic thinking, or a genuinely integrative account. Which of this is strived for appears to depend not only on the context, but more specifically on how this context is interpreted in cultural terms. Interestingly, the likelihood of adopting a supernatural explanation appears to be greater among adults than among children (Astuti and Harris, 2008), suggesting that natural explanations are prevailing early on and are increasingly modified by cultural input.

## Implications of Cognitive Diversity

The studies reviewed in the previous section clearly attest to cognitive diversity not just in the content of cognition, or beliefs more specifically, but on various levels. Processes as basic as perception appear to be modified by the environment in which we grow up, by the language we speak, and by the cultural patterns directing our attention. Cultural tools and practices such as counting sequences and measuring with rulers affect how numerical intuitions are cognitively represented, whether we are able to precisely assess larger quantities, and which difficulties we encounter during mental arithmetic. And on the most explicit level, our cultural background provides us with a set of beliefs, a range of activities that entail diverging degrees of expertise, and interpretative frameworks for the experience we gain in the interaction with our environment and our cohabiters, human and non-human. Far from being superficial differences in ‘just’ the content of cognition, the latter may affect processes as fundamental as categorization, reasoning, and cognitive maturation. Still, the effects on these levels differ in the way in which they unfold.

To begin in reversed order, explanatory frameworks certainly constitute the most direct and straightforward way in which culture exerts its influence. The lion’s share of beliefs we come to hold throughout our lives is picked up as part of our cultural tradition, through explicit teaching or from observing the patterns of behavior in our social environment. Necessarily, some of these beliefs will conflict with each other, but we learn to integrate them, or at least allow them to co-exist, in line with heuristics that we also learn as part of our cultural background. The degree of cultural variability in this respect might therefore seem unsurprising, and yet it goes beyond what we have explicitly learned in that it also encompasses the inferences drawn from it.

As demonstrated for numerical cognition, cognitive processing is also affected by the cultural tools that were designed for certain purposes and that happen to have properties which set constraints, but also generate affordances, thus inevitably affecting the cognitive processes involved in the task at hand. It is thus not by accident that over the last decades, the domain of numerical cognition has contributed substantially to innovative paradigms in cognitive science—including the idea that cognition can be embodied (e.g., Fischer and Brugger, 2011), distributed (Hutchins, 1995; Zhang and Norman, 1995), and extend our mind (De Cruz, 2008)—and has served as an emblematic example of cultural bootstrapping (Miller and Paredes, 1996). Yet, current models of numerical cognition are based on studies with people using a distinct decimal system accompanied by written notation, and are therefore not (yet) able to account for differences that may emerge from such system-specific properties and/or a lack of written notation more generally.

On the basic level of perception and categorization, finally, the influence exerted by culture remains rather subtle, but may be pervasive nonetheless. Here, our cognitive processor is trained by the constant confrontation with information input—either directly from an environment shaped by cultural activities, or indirectly through the spectacles of a linguistic taxonomy. While the latter has been subject to intense research and continuous debate (reviewed in Enfield, 2015), the former has been largely neglected, which is why even the hypotheses on how exactly culture affects perception have remained speculative.

The most important insight from the findings summarized here is that diversity provides highly valuable information, not only in its own right, but also for theoretical advances in cognitive science as it allows one to correct or refine existing models and to reconstruct the emergence and evolution of cognitive phenomena, together with their possible changes over time (Levinson, 2012; Levinson and Gray, 2012). Cognitive processes are tailored to a specific format of representation, which in turn facilitates some processes while hampering others. Scrutinizing these processes irrespective of content or context—which, because they may vary, are conceived of as blurring those findings cognitive science actually strives for—only blurs the insights we obtain (Bender et al., 2015a). Our prospect of approaching a comprehensive understanding of cognition therefore depends crucially on our willingness to take content and cultural variation into account.

## Author Contributions

Both authors have contributed to the work, and approved it for publication.

## Conflict of Interest Statement

The authors declare that the research was conducted in the absence of any commercial or financial relationships that could be construed as a potential conflict of interest.

## Footnotes

1 Please note that the above picture characterizes the tendencies in *mainstream* cognitive psychology and cultural anthropology. Exceptions to these comprise cognitive anthropologists (e.g., D’Andrade, 1995; Kronenfeld et al., 2011) and ethno-linguists (e.g., Lucy, 1992a,b; Gumperz and Levinson, 1996) engaging in cross-cultural studies, cultural psychologists in the tradition of Luria (1976) and Vygotsky (1978) as well as Bruner (e.g., Bruner et al., 1966) and Cole (Cole et al., 1971; Cole and Scribner, 1974) and the school in social psychology evolved around the idea that thinking styles are shaped by the social orientation embedded in broad cultural traditions (reviewed in Nisbett et al., 2001; Varnum et al., 2010; Grossmann and Na, 2014).
